# Emergency carotid artery stenting during endovascular treatment of tandem lesions in acute ischemic stroke: a single-center retrospective study

**DOI:** 10.1186/s42155-026-00712-9

**Published:** 2026-06-11

**Authors:** R. Kaufmann, L. C. van Dijk, W. Stomp, K. F. de Laat, S. F. T. M. de Bruijn, J. R. Piet, S. J. C. Klink, H. van Overhagen

**Affiliations:** 1 Interventional Radiology, Haga Teaching Hospital, Els Borst-Eilersplein 275, The Hague, NL-2545 AA The Netherlands; 2 St. Antonius Hospital, Neurology, Koekoekslaan 1, Nieuwegein, NL-3435 CM The Netherlands; 3 Haga Teaching Hospital, Neurology, Els Borst-Eilersplein 275, The Hague, NL-2545 AA The Netherlands

**Keywords:** Carotid artery thrombosis, Ischemic stroke, Netherlands, Stents, Large vessel occlusion, Tandem lesion

## Abstract

**Background:**

Acute ischemic stroke (AIS) can be caused by multiple factors, including intracranial large vessel occlusion (LVO), severe stenosis or occlusion of the cervical carotid artery, or both (tandem lesion). The optimal treatment strategy for tandem lesions remains unclear. This single-arm retrospective study aims to measure outcomes for tandem lesion patients who were treated with emergency carotid artery stenting (eCAS) during endovascular treatment (EVT).

**Materials and methods:**

Patients who underwent EVT including eCAS for AIS with tandem lesions during the period 2015–2023 at our community teaching hospital in Western Europe were analyzed. Primary outcome was modified Rankin Scale (mRS) at 90-day follow up. Secondary outcomes were mortality within 30 days after stroke, symptomatic intracranial bleeding within 30 days after the stroke, major complications, and angiographic scores.

**Results:**

Of the 600 patients who were treated with EVT, 64 underwent 65 eCAS procedures. Preprocedural National Institutes of Health Stroke Scale (NIHSS) scores of these 64 patients ranged from 2 to 24 (median 13). Expanded treatment in cerebral infarction (eTICI) scores immediately postprocedural were 0 (*n* = 1, 1.5%), 2a (*n* = 6, 9.2%), 2b (*n* = 31, 47.7%), 2c (*n* = 6, 9.2%), or 3 (*n* = 20, 30.8%). In 10.8% (*n* = 7) of eCAS procedures, complications occurred during the procedure and in 18.5% (*n* = 12) complications occurred during the follow-up period. In 9.2% (*n* = 6) of procedures, patients experienced symptomatic intracranial bleeding. Intracranial bleeding was observed more frequently in patients with preprocedural internal carotid artery (ICA) occlusion than stenosis but the difference was not statistically significant.

At 90 days follow-up, in 41.5% (*n* = 27) of eCAS procedures, patients had an mRS score of ≤ 2. Binary logistic regression analysis revealed age and IV thrombolysis as significant predictors for death and age and a pretreatment low NIHSS score for a good functional outcome after 90 days (mRS 0–2). For bleeding complications none of the predictors reached statistical significance.

**Conclusions:**

Emergency carotid artery stenting during EVT for tandem lesions appears feasible with acceptable functional outcomes, despite the occurrence of symptomatic intracranial bleeding in 9% of patients. We must however await the results of randomized controlled trials before firm conclusions about the safety of eCAS can be drawn.

## Introduction

Acute ischemic stroke (AIS) is a major cause of death and disability [1]. AIS can be caused by multiple factors, such as intracranial large vessel occlusion (LVO) or stenosis/occlusion of the cervical internal carotid artery (ICA). Approximately 20 to 30% of all AIS is caused by a LVO. The simultaneous presence of an intracranial occlusion and stenosis/occlusion of the cervical carotid artery constitutes a tandem lesion, which occurs in approximately 15% of LVOs [2, 3].

In the majority of cases tandem lesions are caused by atherosclerotic plaques (60 to 70%), followed by dissections (20% to 30%), with the remainder resulting from more rare causes such as isolated (floating) thrombi without associated high-grade atherosclerotic stenosis, and, more rarely, from radiotherapy-induced stenosis, immune-mediated arteritis, or carotid webs [4]. Patients suffer particularly severe outcomes if they have a tandem lesion [5].

Since a decade, patients suffering from AIS due to an intracranial LVO have the opportunity to undergo endovascular treatment (EVT) [1]. Safety and efficacy have been proven in various randomized controlled trials [6–10] and endovascular treatment has become the preferred choice of care ever since.

In the presence of a tandem lesion, the optimal treatment strategy for the extracranial carotid artery lesions remains undecided; yet, both type and timing of treatment are subject of discussion. Options for treatment comprise of carotid artery stenting (CAS) (emergency stenting or at a later stage), or carotid endarterectomy at a later stage.

Previous papers reported advantages of concurrent CAS during EVT for tandem lesions as follows. Patients only have to undergo one intervention and concurrent treatment immediately reduces the risk of a recurrent LVO due to emboli from the internal carotid artery. Subgroup analysis of the MR CLEAN registry indeed showed that tandem lesion patients who underwent eCAS during EVT had similar functional outcomes as patients undergoing EVT of an intracranial occlusion without eCAS [11]. Combining eCAS and EVT in one session could therefore be feasible without causing additional complications. We cannot, however, neglect the potential risks of CAS in combination with EVT of an intracranial large vessel occlusion. The probable necessity of anticoagulant and/or antiplatelet medication together with the risk of a hyperperfusion syndrome after CAS may cause intracranial hemorrhage. However, this risk and its effect on functional outcome are still debated. In a recent meta-analysis in which 113 of 329 stroke patients with a tandem lesion were treated with eCAS the result showed better functional outcomes and no increase in intracranial hemorrhage after eCAS. Currently randomized controlled studies are underway: for instance, the ongoing CASES study [12].

The aim of our single-arm retrospective study was to assess safety and efficacy of eCAS in the treatment of tandem lesions during EVT in AIS while awaiting the results of randomized studies.

## Materials and methods

### Study type

A retrospective single-arm study was performed in a large community teaching hospital in Western Europe. Patient files from 2015 to 2023 were retrospectively analyzed with regards to baseline characteristics, EVT procedure, and complication rates.

### Ethical approval

Approval was granted by the local medical ethics committee. Anonymized patient data were used. For this type of study formal informed consent is not required.

### Patient selection

Patients were included if they were at least 18 years old and underwent EVT combined with eCAS for tandem lesions. Tandem lesions were defined as a concurrent extracranial stenosis or occlusion of the internal carotid artery and an intracranial large vessel occlusion, displayed on the preprocedural CT angiography (CTA) of the head and neck. Patients with preprocedural dissections were also included in this study.

Inclusion criteria: eCAS was indicated if the cervical part of the carotid artery was stenosed > 50% or occluded on preprocedural CTA. The CTA findings had to be confirmed on angiography at the beginning of the EVT procedure.

Exclusion criteria: clear infarction and/or cerebral hemorrhage on preinterventional non-contrast CT of the brain (NCCT).

### Patients and outcomes

Demographic and clinical data were taken from patient files between January 1, 2015, and December 1, 2023. Baseline patient characteristics included age, gender, risk factors, grade of stenosis, side of the stented carotid artery, and various time metrics (defined as time in minutes between two relevant time points). Onset-to-needle time (stroke onset to administration of intravenous thrombolysis), door-to-needle time (patient admission to the hospital until administration of intravenous thrombolysis), door-to-puncture time (patient admission to the hospital until groin puncture as the start of EVT), puncture-to-recanalization time (groin puncture as the start of EVT until intracranial recanalization), and onset-to-recanalization time (stroke onset until intracranial recanalization).

### Preprocedural CT imaging

All patients underwent a NCCT brain and a CTA of the head and neck. In a later stage (from 2018 on) CT perfusion was added to the CT protocol. In our hospital, we also assessed the presence of collaterals with a separate CT collateral protocol. Preprocedural NCCT was scored using the Alberta Stroke Program Early CT Score (ASPECTS) [13]. This is a 10-point quantitative score used on non-contrast CT scans to assess early ischemic changes in acute middle cerebral artery (MCA) stroke. A score of 10 indicates a normal scan, while 1 point is subtracted for every region showing ischemic changes, with 0 indicating extensive damage. CTA was assessed for the presence of a cervical carotid artery lesion and an intracranial large vessel occlusion. The presence of collaterals was also reported. In the single-phase CTA, we used the scoring system by Tan et al. (0: absent collateral supply to the occluded MCA territory; 1: collateral supply filling ≤ 50% but > 0% of the occluded MCA territory; 2: collateral supply filling > 50% but < 100% of the occluded MCA territory; 3: 100% collateral supply of the occluded MCA territory) [14] and in the multi-phase CTA we used the mCTA collateral score by Menon et al. [15]. A score on a scale of 0 to 5 is given, with 5 being the best and 0 the worst.

CT perfusion analysis consisted of various series assessing brain perfusion (including cerebral blood volume, cerebral blood flow, time to peak (Tmax), mean transit time, and time to drain). Analysis of these series attempts to differentiate salvageable ischemic brain tissue (the penumbra) from the irrevocably damaged infarcted brain (the infarct core). A mismatch ratio > 1.8 (or in some protocols > 1.2), with a core volume < 70 ml, indicates a large, clinically significant area of at-risk brain tissue, guiding patient selection for late-window endovascular thrombectomy (6–24 h). In addition, a Potential Recuperation Ratio (PRR) was calculated to measure the relative size of the salvageable penumbra compared to the total ischemic area (penumbra + core). A higher PRR indicates a better prognosis and greater potential for recovery.

**Fig. 1:**
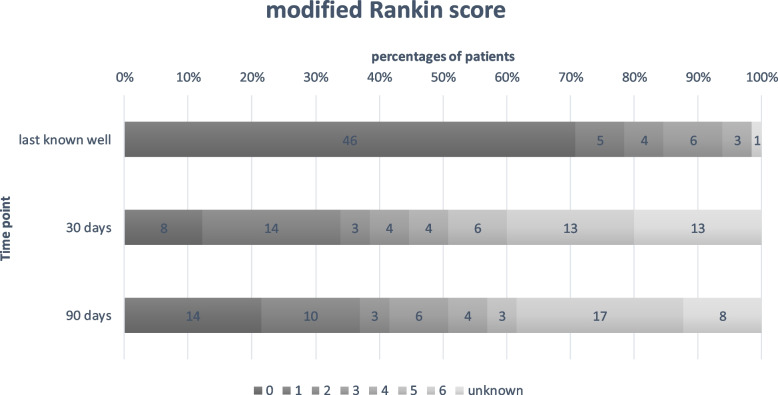
Modified Rankin Scale scores premorbide, 30 days, and 90 days for patients who received eCAS procedure

Primary outcome was the functional outcome of the patient as measured with the modified Rankin Scale score at 90 days after the stroke. The modified Rankin Scale (mRS) is a 7-point ordinal scale (0–6) that quantifies global disability and dependence in activities of daily living after stroke; higher scores indicate worse outcome, with 6 representing death. It is the most widely used functional outcome measure in stroke trials and routine clinical practice to categorize post-stroke disability and to define “good” versus “poor” outcome (commonly mRS 0–2 vs 3–6). These data were obtained prospectively, since this is mandatory for participation in the national acute stroke audit quality register. Data are shown in Fig. 1.

Secondary outcomes were mortality within 90 days after the stroke, symptomatic intracranial hemorrhage within 30 days after the stroke, major complications (i.e., symptomatic intracranial hemorrhage, access site complications, other complications such as stent thrombosis), and angiographic score. Only symptomatic patients (i.e., clinical deterioration) received postprocedural imaging. Procedure characteristics of interest included stent type, stent diameter, stent length, EVT location, and preprocedural and periprocedural medication.

### Intervention

Treatment was started as soon as possible after clinical diagnosis and CT. Patients were transferred from the emergency department and CT to the intervention suite. Under ultrasound guidance, local anesthesia was given (10 mL Lidocaine 1%), and an 8Fr sheath was placed in the common femoral artery. The cervical part of the internal carotid artery (ICA) was catheterized using a standard 4 F or 5 F neuro-angiography catheter. Either by using the telescope technique or by using a Hi-Torque Supra Core 0.035″ exchange wire (Abbott, Illinois, USA), an 8 F guiding catheter (Neuron Max 088, Penumbra Inc., Alameda, USA) was placed in the distal part of the common carotid artery. Emergency carotid artery stenting (eCAS) was performed by placing a self-expanding stent over a 0.014″ guidewire (Balance Middleweight Universal II (Abbott, IL, USA) or Glidewire Advantage (Terumo Interventional Systems, Tokyo, Japan)). Stenting was performed by placement of either a Carotid Wallstent (Boston Scientific, Minneapolis, USA) or a Precise Pro RX stent (Cordis, Miami Lakes, USA).

Pre- and/or post-dilation were performed at the operator’s discretion. Intracranial arterial treatment of the intracranial occlusion consisted of thrombosuction, thrombectomy, or a combination of both. Intracranial thrombus removal was performed using an aspiration device (Red Reperfusion Catheter (Penumbra Inc., Alameda, USA) or MicroVention Sofia (Terumo Interventional Systems, Tokyo, Japan)) and/or stent retriever (Solitaire (Medtronic, Dublin, Ireland). At the end of the procedure, the groin was closed with an 8Fr Angio-Seal® VIP vascular closure device (Terumo Interventional Systems, Tokyo, Japan).

### Medication

Medication of patients was assessed at the arrival in the hospital. In the case of intravenous thrombolysis, patients received 0.9 mg/kg Actilyse (alteplase) before the EVT procedure. Antithrombotic strategy during and after the procedure: all patients received one bolus of 5000 international units (IU) of heparin intra-arterially directly following the placement of the femoral artery sheath. After the procedure, a loading dose of 300 mg Clopidogrel and Aspirin 80 mg and on the next day Clopidogrel 75 mg daily + aspirin 80 mg daily was started if no signs of intracranial hemorrhage were present. Additional use of medication during the procedure was registered.

### Clinical assessment

The severity of the stroke was assessed using National Institutes of Health Stroke Scale scores (NIHSS). Recanalization was measured using the expanded treatment in cerebral infarction (eTICI) score. Assessment of the angiography images was performed independently in a blinded fashion by two interventional radiologists.

### Statistical analysis

Data were collected using Castor EDC and Excel (Microsoft Office). Statistical analysis was performed using IBM SPSS Statistics, version 28.0.1.0(142). Continuous data were reported as median and interquartile range. Categorical variables were reported as number of events and percentages. Proportions and percentages are expressed with the number of procedures instead of the number of patients as the denominator. Binary logistic regression analysis was performed analyzing the influence of age, NIHSS, administration of IVT, and occlusion type on various patient outcomes. Binary dependent outcomes were bleeding complications (yes/no), mRS at 90 days 0–2 vs 3–6, and death (yes/no).

## Results

### Patient population

During 2015–2023, 600 patients underwent EVT. Of these 600 patients, 64 patients underwent 65 eCAS procedures (Table 1) with the earliest procedure on March 19, 2015, and the latest on July 29, 2023.

**Table 1:** Baseline characteristics of 65 patients who underwent eCAS procedures

Baseline characteristics	Patients (*N* = 65)
Age (years)	
- Mean	71.2
- Range	52–99
Male, *n* = (%)	43 (67.2%)
Time metrics	
- Onset-to-needle time	15 to 260 min (mean 88.7 min; SD 48.7)
- Door-to-needle time	11 to 140 min (mean 24.7 min; SD 22.2)
- Door-to-puncture time	8 to 160 min (mean 71.6 min; SD 37.0)
- Puncture-to-recanalization time	15 to 198 min (mean 62.6 min; SD 40.3)
- Onset-to-recanalization time	96 to 340 min (mean 192.9 min; SD 73.8)
Risk factors, *n* (%)	
- Hypertension	40 (62.5%)
- Hypercholesterolemia	19 (29.7%)
- Diabetes mellitus	17 (26.6%)
- Myocardial infarction	9 (14.1%)
- Cerebrovascular accident (stroke) in history	11 (17.2%)
- Atrial fibrillation	15 (23.4%)
- Current smoker	21 (32.8%)
Grade of stenosis (% of procedures)	
- Occlusion	29 (44.6%)
- Moderate (50–70%)	7 (10.8%)
- Severe (> 70%)	12 (18.5)
- Near occlusion	11 (16.9%)
- Dissection preprocedural	3 (4.6%)
- Dissection during procedure	3 (4.6%)
Stenting performed in internal carotid artery (%)	
- Left side	32 (49.2%)
- Right side	33 (50.8%)

The patients who underwent eCAS procedures were 43 men (67%) and 21 women (33%) with a mean age of 71.2 years (range 52–99 years).

### Time metrics

Onset-to-needle time ranged from 15 to 260 min (mean 88.7 min; SD 48.7). Door-to-needle time ranged from 11 to 140 min (mean 24.7 min; SD 22.2). Door-to-puncture time ranged from 8 to 160 min (mean 71.6 min; SD 37.0). Puncture-to-recanalization time ranged from 15 to 198 min (mean 62.6 min; SD 40.3). Onset-to-recanalization time ranged from 96 to 340 min (mean 192.9 min; SD 73.8).

### Preprocedural CT imaging

Images of the preprocedural NCCT were available for all but one patient. We found no intracranial hemorrhage and in 44 patients no early signs of infarction were present (ASPECTS score 10). In the remaining 20 patients, we found ASPECTS scores of 6 (*n* = 1; 1.5%), 7 (*n* = 2; 3.1%), 8 (*n* = 2; 3.1%), and 9 (*n* = 15; 23.1%).

Preprocedural CTA revealed intracranial LVOs at the following sites: Carotid-T *n* = 1 (1.5%), Carotid-T/M1 *n* = 1 (1.5%), M1 *n* = 35 (53.8%), M1/M2 *n* = 2 (3.1%), M2 *n* = 20 (30.8%), M2/M3 *n* = 1.5%, M3 *n* = 3 (4.6%), A1/M1 *n* = 1 (1.5%), and A2 *n* = 1 (1.5%).

The collateral score based on single-phase CTA ranged from 1 to 3 with a mean of 2 and the collateral score based on the multi-phase CTA ranged from 2 to 5 with a mean of 4.

CTA perfusion parameters were available for 38 patients (58.5%). In 9 cases, only the images were available for assessment, no quantitative analysis was performed in these patients. In the remaining patients, we assessed the infarct core ranging from 0.70 to 32.6 cm^3^ (mean 16.3 cm^3^), penumbra ranging from 26.3 to 204.00 cm^3^ (mean 78.5 cm^3^), and PRR ranging from 46.6 to 99.7% (mean 82.7%). Perfusion mismatch ratio ranged from 1.87 to 292.43 (mean 19.2).

### Medication

Thirty-four (53.1%) patients were on some kind of platelet inhibitor therapy or anticoagulant therapy. Intravenous thrombolysis (0.9 mg/kg Alteplase) was administered prior to the procedure in 37 of 65 (57%) eCAS procedures.

Local administration of 10 mL Lidocaine 1% was performed in all patients before arterial access was obtained. Then 5000 IU heparin was administered intra-arterially in all patients immediately after inserting the sheath into the common femoral artery. If the procedure lasted more than an hour (*n* = 8), patients occasionally received an additional dose of 3000 or 5000 IU heparin after an hour. In 15.4% of procedures (*n* = 10), it was not recorded which dose of heparin the patient received. In eight procedures (12.3%), patients received Atropine (0.25–2 mg) iv. Twelve patients (18.5%) received Nimodipine (1–2 mg i.a.). Fourteen patients (21.5%) received Labetalol due to perprocedural hypertension.

### Carotid artery stenting

Stenting was performed using 53 Carotid Wallstents (Boston Scientific, Minneapolis, USA) and 12 Precise Pro RX stents (Cordis, Miami Lakes, USA). The median stent diameter was 7 mm (range 4–9) and median stent length was 30 mm (range 30–60).

Additional EVT was performed in 54 procedures, at the following locations: carotid T (*n* = 3), M1 (*n* = 28), M1/M2 (*n* = 3), M2 (*n* = 18), M3 (*n* = 1), A2 (*n* = 1). One patient had a remaining M3 occlusion, but EVT was not performed because it was considered too risky. Eleven intracranial lesions present on preprocedural CTA were resolved after eCAS without intracranial thrombectomy. Postprocedural medication consisted of a loading dose of 300 mg clopidogrel and aspirin 80 mg on the stroke ward immediately after the procedure if there were no signs of intracranial hemorrhage. The next day followed by clopidogrel 75 mg daily + aspirin 80 mg daily in the absence of signs of intracranial hemorrhage.

### Clinical outcomes

Preprocedural NIHSS scores ranged from 0 to 24 (median 13). Data were available in the electronic patient files for all patients. After 30 days, in 25 (38.5%) procedures patients had an mRS score of 2 or below. After 90 days, in 27 (41.5%) procedures patients had an mRS score of 2 or below.

Emergency carotid artery stenting was performed in case of a significant (> 50%) atherosclerotic internal carotid artery stenosis (*n* = 30 (46.2%)), internal carotid artery occlusion (*n* = 29 (44.6%)), or internal carotid artery dissection (*n* = 6 (9.2%); 3 of 6 occurred during EVT) (also see Table 1). Carotid artery stenting was performed prior to intracranial revascularization in all procedures; except in one patient who suffered from a dissection during the EVT procedure.

### Technical and safety outcomes

Stenting was performed in the left (*n* = 32 (49.2%)) or right carotid artery (*n* = 33 (50.8%)). Predilation and postdilation of the internal carotid artery were performed in 50.8% (*n* = 33) and 53.8% (*n* = 35) of procedures, respectively. Data regarding the PTA balloon size used for predilation of the internal carotid artery were available in 32 (49.2%) procedures. In 36.9% (*n* = 24) of procedures predilation was performed with a 3-mm PTA balloon, in 3.1% (*n* = 2) with a 4-mm and in 7.7% (*n* = 5) with a 5-mm balloon. In 1.5% (*n* = 1) of procedures a 7-mm balloon was used.

Data regarding the size of the PTA balloon used for postdilation of the internal carotid artery were available for 53.8% (*n* = 35) of procedures. In 1.5% (*n* = 1) of procedures postdilation was performed with a balloon diameter of 4 mm, 1.5% (*n* = 1) of 4.5 mm, 49.2% (*n* = 32) of 5 mm, and 1.5% (*n* = 1) of 7 mm.

eTICI scores immediately postprocedural were 0 (*n* = 1, 1.5%), 2a (*n* = 6, 9.2%), 2b (*n* = 31, 47.7%), 2c (*n* = 6, 9.2%), or 3 (*n* = 20, 30.8%). One patient received no eTICI score because there was no completion angiography.

### Periprocedural complications

Seven complications occurred during the procedure in 7 patients (10.8%). These included dissection of the internal carotid artery (*n* = 3, 4.6%), access bleeding (*n* = 1, 1.5%), extravasation from M2 segment (*n* = 1, 1.5%), material too short to reach remaining thrombus in M1 segment (*n* = 1, 1.5%), and solitaire stent retriever trapped in a cervical ICA stent (*n* = 1, 1.5%).

During the entire follow-up period, complications occurred in 12 procedures (18.5%), including symptomatic intracranial hemorrhage (*n* = 6 (9.2%)), access site complications (*n* = 2 (3.1%)), intracranial (M2) re-occlusion (*n* = 1 (1.5%)), epistaxis (*n* = 1 (1.5%)), severe headache (*n* = 1 (1.5%)), and an epileptic insult (*n* = 1 (1.5%)). During the 90-day follow-up period, no patients suffered from in-stent thrombosis.

Subgroup analysis showed that the number of symptomatic intracranial bleeding complications in patients with a preprocedural ICA occlusion was higher (5 out of 29 procedures (17.2%)) than in patients with an ICA stenosis (1 out of 30 procedures (3.3%)). No statistically significant difference was found between these two subgroups.

Binary logistic regression analysis revealed age and IV thrombolysis as significant predictors for death and age and NIHSS score for a good functional outcome after 90 days (mRS 0–2). For bleeding complications none of the predictors reached statistical significance. Also see Table 2.

**Table 2:** Binary logistic regression analysis

Outcome measures	Variable	*P*-value	Odds ratio	Confidence interval
mRS at 90 days (0–2 vs 3–6)	Age	*P* = 0.002	1.143	1.050–1.245
	NIHSS	*P* = 0.016	1.171	1.029–1.333
Bleeding complications	No significant predictors			
Death	Age	*P* = 0.019	1.084	1.014–1.160
	IV thrombolysis	*P* = 0.042	0.238	0.060–0.950

During the 90-day follow-up period, 16 out of 64 patients (25%) deceased, due to pneumonia (*n* = 7; 10.9%), bleeding complications (*n* = 3; 4.7%), cardiac failure (*n* = 1; 1.6%), or unknown causes after discharge from the hospital (*n* = 5; 7.8%).

## Discussion

Acute ischemic stroke in patients with tandem lesions often leads to poor clinical and functional outcomes without treatment [16]. Intracranial EVT of LVOs substantially improves clinical outcomes. The best strategy to treat the cervical carotid artery lesion is subject to many studies currently. We assessed concurrent eCAS. Alternative treatment strategies comprise of PTA only [17], postprocedural best medical treatment [18], or surgical carotid endarterectomy or carotid artery stenting within two weeks after the initial event [19]. In our institution, we perform elective carotid artery stenting since the year 2000 and we adopted “emergency carotid artery stenting on the way up” directly when we started with EVT in the treatment of stroke.

In our current study, we found that concurrent emergency carotid artery stenting (eCAS) and endovascular treatment (EVT) in patients with an acute ischemic stroke due to a tandem lesion led to acceptable functional outcomes, despite symptomatic intracranial bleeding complications in 9.2% of patients. In comparison, a recent study by Simon et al. reported 20% intracranial bleeding complications. From this report it is unclear whether these are only symptomatic or all intracranial bleeding complications [3]. Another study by Cavalcante et al. reported symptomatic intracranial bleeding complications in 6.3% of patients, which seems more consistent with our findings [20].

In our population, heparin was administered to prevent acute stent thrombosis despite the fact that the administration of heparin in stroke patients is regarded as controversial in the literature. In our study, in 9.2% of procedures patients suffered from symptomatic intracranial bleeding complications, somewhat lower than the 13–13.5% of patients with intracranial bleeding after receiving heparin in previous studies [21, 22]. In a meta-analysis evaluating the effects of heparin administration during EVT, Jazayeri et al. reported higher risk of cerebral hemorrhage after the administration of heparin, especially in combination with intravenous thrombolysis [23]. Although iv thrombolysis was not associated with increased intracranial bleeding in our study, it was associated with a worse clinical outcome. These findings suggest that there is a fine balance between risk and benefit of heparin administration during endovascular stroke treatment. Currently performed randomized trials studying eCAS in stroke should provide more solid evidence how to manage thrombosis and bleeding risks around these interventions.

In our study, the risk of a symptomatic intracranial bleeding complication was higher after eCAS for an occlusion than for a stenosis but the difference did not reach statistical significance. This does not seem surprising since an occluded carotid artery will reduce blood flow toward the brain even more than a stenosis. Absence of blood flow may decrease the patient’s own thrombolytic system. This has been shown by us in a previous report where intracranial arterial occlusion on CTA in patients with (pseudo) occlusion of the cervical internal carotid artery disappeared after carotid recanalization and stenting, probably because of the patient’s own thrombolytic system [24]. Reopening the vessel in case of occlusion and loss of autoregulation may indeed increase the risk of bleeding.

A lower age and a lower pretreatment NIHSS score were associated with a better clinical outcome: this could be explained by the generally better condition of these patients.

When stenting a cervical carotid artery in patients undergoing EVT we prefer to use a closed-cell design. The chance that the thrombectomy device gets caught behind the struts of the stent is likely to be smaller than when using an open-cell design. Another strategy to avoid this problem can be to insert the guiding catheter above the stent but this is not feasible in all patients.

Taking everything into consideration, a recent review article of Krothapalli et al. could not provide definitive answers to questions regarding the role of eCAS in the treatment of tandem lesions despite reports of encouraging results. Current randomized trials such as CASES, EASI-TOC, TITAN, PICASSO, and START are expected to provide us with scientifically substantiated advice for the treatment of these complex patients in the future [25].

### Strengths and limitations

The strength of our study is that we analyzed 65 procedures in one center performed by four experienced interventional radiologists, excluding learning curve bias.

Limitations are that our retrospective, single-center cohort study included cases with both atherosclerotic stenosis and occlusion and dissection, potentially introducing a bias. There were no data available on symptomatic bleeding complications in patients with an LVO only.

## Conclusion

Emergency carotid artery stenting during EVT for tandem lesions appears feasible with acceptable functional outcomes, despite the occurrence of symptomatic intracranial bleeding complications in 9.2% of patients. We must, however, await the results of randomized controlled trials before firm conclusions about the safety of eCAS can be drawn.

## Data Availability

The data are available from the corresponding author upon reasonable request.

## Abbreviations

AIS: Acute ischemic stroke
CAS: Carotid artery stenting
CTA: CT angiography
eCAS: Emergency carotid artery stenting
eTICI: Expanded treatment in cerebral infarction
EVT: Endovascular treatment
ICA: Internal carotid artery
IU: International units
LVO: Large vessel occlusion
mRS: Modified Rankin Scale
NCCT: Non-contrast computed tomography
NIHSS: National Institutes of Health Stroke Scale scores
PTA: Percutaneous transluminal angioplasty
